# Mortality & recurrent seizure risk after new-onset seizure in HIV-positive Zambian adults

**DOI:** 10.1186/s12883-018-1205-2

**Published:** 2018-12-07

**Authors:** Melissa A. Elafros, Brent A. Johnson, Omar K. Siddiqi, Jason F. Okulicz, Izukanji Sikazwe, Christopher M. Bositis, Michael J. Potchen, Igor J. Koralnik, William H. Theodore, Lisa Kalungwana, Gretchen L. Birbeck

**Affiliations:** 1 Department of Neurology, Johns Hopkins Hospital, Sheik Zayed Tower, Room 6005, 1800 Orleans Street, Baltimore, MD 21287 USA; 2 Department of Biostatistics and Computational Biology, University of Rochester, 265 Crittenden Boulevard, CU 420-630, Rochester, NY 14642-0630 USA; 3 Global Neurology Program, Division of Neuroimmunology, Department of Neurology, E/CLS 1017B Beth Israel Deaconess Medical Center, 330 Brookline Avenue, Boston, MA 02215 USA; 4 Department of Internal Medicine, University of Zambia School of Medicine, Lusaka, Zambia; 5 Infectious Disease Service, Brooke Army Medical Center, 3851 Roger Brooke Dr., Fort Sam Houston, San Antonio, TX 78234 USA; 6 Center for Infectious Disease Research in Zambia, 5032 Great North Road, P.O. Box 34681, Lusaka, Zambia; 7 Greater Lawrence Family Health Center, 34 Haverhill St, Lawrence, MA 01841 USA; 8 Neuroradiology Division, Department of Imaging Sciences, University of Rochester Medical Center, 601 Elmwood Ave, Box 648, Rochester, NY 14642 USA; 9 Department of Neurological Sciences, Rush University Medical Center, 1725 W. Harrison Street, Suite 1106, Chicago, IL 60612 USA; 10 Clinical Epilepsy Section, National Institute of Neurological Disorders and Stroke, NINDS NIH Building 10 Room 7D-43, Bethesda, MD 20892 USA; 11 Department of Psychology, University of Zambia, P.O. BOX 32379, 10101 Lusaka, Zambia; 12 Epilepsy Division, Department of Neurology, University of Rochester School of Medicine & Dentistry, 265 Crittenden Blvd, CU420694, Rochester, NY 14642–0694 USA; 13 Epilepsy Care Team, Chikankata Hospital, Private Bag S2, Mazabuka, Zambia

## Abstract

**Background:**

Recurrent seizure risks in HIV-positive people with new-onset seizure are largely unknown, making it challenging to offer optimal recommendations regarding antiepileptic drug (AED) initiation. Existing outcomes data is limited, and risk factor identification requires a diagnostic assessment, which is often unavailable in regions heavily effected by HIV, like sub-Saharan Africa.

**Methods:**

HIV-positive Zambian adults with new-onset seizure were enrolled in a prospective cohort study to determine seizure recurrence and risk factors for recurrence. Seizure etiology was evaluated, and recurrent seizures and medication usage were assessed during clinic visits. Due to unexpectedly high mortality rates, predictors of death were evaluated using proportional hazards with Gray’s test to compare cumulative incidence functions for recurrent seizure across groups adjusting for the competing outcome of death.

**Results:**

95 patients were enrolled (mean age 37 years, 43% female, 83% with Karnofsky > 50) and followed for a mean of 293 days (median 241 (IQR: 29–532)). At presentation, 50 (53%) were in *status epilepticus*. The majority (91, 85%) had advanced HIV disease and 65 (68%) were not on combined antiretroviral therapy (cART). After extensive workup, seizure etiology remained unknown in 16 (17%). Average time to cART initiation after enrollment was 61 days. During follow up, 37 (39%) died and 23 (24%) had recurrent seizure. Most deaths (25/37, 68%) occurred in the first 60 days post-index seizure. Individuals with advanced HIV were more likely to die (HR: 19.1 [95% CI: 1.1–333.4]) as were those whose seizure etiology remained unknown (HR: 2.2 [95% CI: 1.1–4.4]). Among participants that survived from enrolment to the end of data collection on 10 May 2013 (*n* = 58), 20 (34%) experienced recurrent seizures.

**Conclusions:**

New-onset seizure among HIV-positive Zambian adults is associated with high mortality despite good functional status prior to presentation. Advanced HIV infection and failure to identify an underlying seizure etiology are associated with greater mortality. Recurrent seizures occur in over a third of survivors within only 2 years of follow-up. This provides evidence to support AED initiation after first seizure in HIV-positive individuals with advanced HIV disease at the time of presentation though the risks of AED-cART interactions remain a concern and warrant further study.

## Background

HIV infection is a recognized seizure risk factor [1]. Hospital-based studies suggest convulsions are a presenting symptom in 2–13% of HIV-positive adults regardless of treatment status with combination antiretroviral therapy (cART) [2–4]. Often, no underlying seizure etiology is identified despite a thorough workup including electroencephalogram (EEG), neuroimaging, and cerebrospinal fluid (CSF) analysis and HIV’s effects on the central nervous system (CNS) is the presumed cause [5]. Unfortunately, very limited diagnostic capacity is available in resource-limited settings and existing data on seizure etiology in HIV-positive individuals is frequently limited to a small subset of individuals with a complete workup limiting generalizability [2–4, 6].

Retrospective [3, 4, 6] and prospective [2, 7–10] studies have examined new-onset seizure etiology in HIV-positive individuals, but few assess long-term health outcomes, including mortality and the risk of recurrent seizure [11]. Follow up is often incomplete [4, 12] or for unknown duration [3, 5, 9, 10]. Two U.S. studies conducted prior to cART and one Spanish study after cART tracked patients on average for 5 months [4, 6, 7]. Data from India showed that 65% of HIV-positive inpatients with new-onset seizure experienced recurrent seizure, however, it is unclear whether these observed seizures occurred during or after the initial hospitalization [2]. Since initiating antiepileptic drugs (AEDs) with cART may adversely affect HIV virologic suppression due to enzyme-induction, predispose to AED- and cART-associated toxicity, and decrease overall medication adherence due to increased pill burden, accurately characterizing the need for chronic AED treatment in this population is imperative, particularly in resource-limited settings where enzyme-inducing AEDs continue to be the mainstay of epilepsy care [13, 14].

We are conducting a prospective cohort study of HIV-positive individuals presenting with new-onset seizure in Zambia to determine seizure etiology and follow the cohort for relevant outcomes, including seizure recurrence and death [15]. Here, we examine seizure recurrence and death among a subset of patients enrolled in Phase I of our cohort study to summarize the timeline for repeated seizure events while properly accounting for the competing risk of death.

## Methods

The Cohort for HIV-Associated Seizures and Epilepsy (CHASE) Study is an ongoing prospective observational study that enrolled urban adult participants from 8 August 2011 to 10 May 2013. HIV-infected Zambian adults presenting to the University of Zambia’s University Teaching Hospital (UTH) inpatient medical wards and outpatient HIV clinic and to Chreso Ministries ART clinic, a faith-based nongovernmental clinic in Lusaka, were assessed for eligibility. A study nurse went bed-to-bed in the UTH emergency room and inpatient wards to assess admitted patients while a study investigator screened all patients presenting to UTH’s HIV clinic for eligible participants [15]. One of the investigators confirmed each patient’s eligibility and informed consent was obtained from the patient or health care proxy in English or a local language (Nyanja or Bemba) by a trained Zambian nurse.

Eligible participants included patients ≥18 years with documented HIV-infection, presenting within 2 weeks of new-onset seizure. Patients with a history of seizure other than childhood febrile seizures were excluded. To facilitate follow up, participants received their HIV care through the Adult Infectious Disease Centre (AIDC), which includes UTH’s adult HIV clinic. All subjects recruited in Phase I of the CHASE Study are included in this outcomes assessment study.

### Measures at enrolment

The following was collected via structured interview: age; gender; presenting clinical symptoms; and past medical history. Past medical history included seizure-related risk factors and HIV history, which captured duration of diagnosis and treatment. A neurologist characterized seizure type according to Fisher et al’s 2017 operational classification of seizure types using patient and/or witness descriptions of the event or a focal neurologic exam at enrollment [16]. Seizure severity was determined using duration of the index seizure based on clinical report. Status epilepticus included tonic-clonic seizures lasting more than 5 minutes or focal seizures lasting more than 10 minutes [17]. Consciousness at the time of enrolment was characterized with the Glasgow Coma Scale [18]. The WHO Clinical Staging Criteria were used to stage HIV infection [19]. Etiologic assessment included serum testing for: CD4^+^ T-cell count; sodium; glucose; malaria infection using the *P. falciparum* rapid diagnostic antigen test; rapid plasma reagin test for syphilis and, if needed, a confirmatory Treponema pallidum hemagglutination assay. Among patients who consented to a lumbar puncture, CSF testing included: cell count and differential; glucose; total protein; gram stain; Venereal Disease Research Laboratory (VDRL) testing for neurosyphilis; and cryptococcal antigen testing. DNA PCR analysis included: *Mycobacterium tuberculosis*; Epstein-Barr virus (EBV); JC virus; varicella zoster virus; cytomegalovirus; herpes simplex virus type 1; herpes simplex virus type 2; and *Toxoplasma gondii* [15]. Patients who did not consent to a lumbar puncture had serum cryptococcal antigen testing.

Patients sufficiently stable for transportation to the outpatient EEG lab completed a 16-channel EEG using Biologic CEEGgraph equipment. EEGs were performed in accordance with the American Clinical Neurophysiology Society Guidelines using silver-silver chloride electrodes applied with collodion using the international 10–20 electrode placement [20]. Filters were set at 1 and 70 Hz and impedances were below 10 k-ohms. EEGs were interpreted by two neurologists with EEG fellowship training [21, 22]. EEG findings, in addition to patient/witness descriptions and physical exam findings, were used to identify any seizure focality. Neuroimaging was obtained on patients who either had it as part of their routine clinical care or, whose seizure etiology remained unknown after CSF evaluations [23]. All results were shared with the patient’s inpatient and outpatient providers.

Underlying seizure etiology was independently classified into the following categories by two investigators blinded to outcome with external consensus mediation, as needed. Categories delineated post hoc included: CNS OI; other infection such as bacterial meningitis, malaria, or syphilis; structural lesion on neuroimaging; hyponatremia; and unknown. A serum sodium < 135 mmol/L was considered hyponatremia, which was further classified as either primary or secondary [15]. More than one possible underlying seizure assignment was permitted.

### Measures during follow-up

Patients were followed through their hospitalization and, after discharge, via the AIDC. AIDC providers directed all treatment-related decisions. Patients were followed until death, withdrawal, or closure on 18 December 2013. Reminder phone calls and a transport reimbursement of 20 kwacha (~US $4.00) were provided to participants to assist with transportation costs to attend the AIDC appointments. The study facilitated transportation for patients not well enough to use public transportation. Patients were also offered rescheduling assistance for missed appointments. If a patient could not be located, a research assitant and/or study investigator attempted to trace participants using phone numbers patients provided during enrolment or in their clinic file. These phone calls continued once a week until the patient was located, staff were informed the patient had died, the patient withdrew, or the study closed.

At each encounter with patients or close contacts, seizure recurrence data and major medical events, including death, were collected. Among participants experiencing recurrent seizure, the number, date, and a description of each seizure were recorded. In the event of death, circumstances surrounding the event were obtained from a close contact, specifically to determine whether any seizures occurred around the time of death. Data regarding medication use and changes was abstracted from patient files at the time of interview and confirmed by patients.

### Statistical analysis

Baseline demographic and clinical data, seizure etiologies, and follow up data, including duration of follow up and medication changes, were summarized using frequencies and means. The primary outcomes of interest were seizure recurrence and death. Proportional hazards models were used to evaluate clinical and demographic predictors of death. Gray’s method, which accounts for a competing event when comparing the cumulative incidence of an event across risk factors, was used to compare the incidence for recurrent seizure during follow up based on clinical and demographic risk factors, while controlling for the competing risk of death. Characteristics associated with death and seizure recurrence were then examined using the entire study cohort. A subsequent sensitivity analysis was performed using only patients who had CSF available for DNA PCR and, thus, had a complete laboratory workup. All analyses were performed using SAS 9.4 (SAS Institute, Cary, North Carolina). A *p*-value of < 0.05 was considered statistically significant.

### Ethical approval

Ethical approval was obtained from the University of Zambia’s Biomedical Research Ethics Committee (UNZA BREC) as well as Michigan State University’s Biomedical Institutional Review Board (MSU BIRB). All participants or their proxies provided written, informed consent. If a proxy provided initial consent, patient consent was obtained once the patient was well enough to provide it.

## Results

### Baseline

Ninety-five patients (43% female, mean age 36.9 years, SD 10.2 years) participated in the CHASE Study. All were black Zambians. Seventy-nine (83%) patients had a Karnofsky Scale Score > 50 at enrolment. There was no difference in gender (*p* = 0.61), age (*p* = 0.40), presenting seizure severity (*p* = 0.15), or CD4^+^ T-cell count at enrolment (*p* = 0.55) between patients with a Karnofsky Scale Score > 50 and those with a Karnofsky Scale Score < 50.

As shown in Table 1, most patients (78%) presented with tonic-clonic seizures, 33 (35%) with focal onset. Seizure type could not be classified for six (6%) participants. 43 (45%) participants with tonic clonic seizures and 6 (6%) participants with focal clonic seizures met criteria for *status epilepticus*. 54 (57%) had multiple seizures prior to presenting for care, of which 31 (31/54, 57%) had multiple seizures within 24 h. Eleven (12%) patients had an immediate family member with epilepsy; two (2%) had a prior head injury. The majority (85%) of study participants had advanced HIV infection (WHO Stage III or IV) at enrolment. Among those able to provide information regarding their HIV history (*n* = 85), the index seizure led to HIV diagnosis among 46 (54%) patients and, thus, most participants were cART-naïve. Among those on cART at enrolment, 27/32 (84%) were on their initial cART regimen, which in this setting includes a non-nucleoside reverse transcriptase inhibitor, often efavirenz (19/27; 70%). The remaining five patients had failed their initial regimen and had been switched to a regimen containing a protease inhibitor.

**Table 1:** Demographics and clinical characteristics of HIV-positive adults with new-onset seizure (*n* = 95)

*Demographic*	
Gender, female n (%)	41 (43)
Age, mean (SD)	36.9 (10.2)
Family history of epilepsy n (%)	11 (12)
History of head injury n (%)	2 (2)
History of severe malaria n (%)	6 (6)
History of meningitis/encephalitis n (%)	7 (7)
History of coma n (%)	1 (1)
History of opportunistic infection (*n* = 94)^a^ n (%)	5 (5)
History of stroke (n = 94) ^a^ n (%)	4 (4)
*Clinical*	
Seizure type n (%)	
Focal clonic	13 (14)
Focal onset to bilateral tonic-clonic	33 (35)
Tonic clonic	1 (1)
Unknown onset bilateral tonic-clonic	42 (44)
Unclassified	6 (6)
Presenting seizure severity n (%)^b^	
*Status epilepticus*	50 (53)
Glasgow Coma Score, mean (SD)	14.3 (1.9)
Karnofsky score < 50 at enrollment n (%)	16 (17)
WHO clinical stage n (%)^a^	
I or II	12 (13)
III or IV	81 (85)
Current cART use n (%)	
Yes, less than a year	13 (14)
Yes, more than a year	17 (18)
No, defaulted	7 (7)
No, never	58 (61)
CD4^+^ T-cell count at enrolment, mean cells/mm^3^(*n* = 89) ^a^ (SD)	179 (185)
*Underlying seizure etiology* ^*c*^	
CNS opportunistic infection n (%)	21 (22)
Other infection n (%)	8 (8)
Structural lesion n (%)	25 (26)
Hyponatremia (*n* = 91) n (%)	47 (49)
Likely secondary to another etiology	17/47 (36)
Unknown, n (%)	20 (21)

Data is presented as number (%), mean (SD), or median (IQR), as appropriate. *WHO* World Health Organization, *cART* combination antiretroviral therapy, *CNS* central nervous system. ^a^Patient unable to provide information. ^b^Missing data for 6 participants ^c^More than one seizure etiology possible

Eighty-one (85%) participants had CSF collected as part of their workup. There were no significant demographic or clinical differences between patients who underwent a lumbar puncture and those who did not, including time to death (*p* = 0.95). Sixty-one patients (61/81; 75%) had sufficient CSF collected for DNA PCR testing. Eighty-one (85%) participants had an EEG. Of the remaining 14 patients, 12 (86%) died before EEG could be completed. Fifty-five (55/81; 68%) EEGs were abnormal. 23 (33%) had background slowing, 27 (34%) had generalized slowing, and 16 (20%) had focal slowing. Nine (11%) had interictal focal discharges and four (5%) had interictal general discharges. Six (8%) patients had seizures captured during EEG. Sleep architecture was captured on two EEGs. Forty (42%) participants had neuroimaging (38 CT, 2 MRI), of which 70% had an abnormality. White matter abnormalities, primarily vasogenic edema, were the most common abnormality detected (24/40; 56%). Some of these findings have been reported elsewhere in papers dedicated to these specific risk factors [22, 23].

Twenty-one (22%) patients had a CNS OI, eight (10%) had another identified infection (bacterial meningitis (4), malaria (1), and neurosyphilis (3)), and 47 (49%) were hyponatremic (Table 1). Of those with neuroimaging (*n* = 40), 25 (63%) had a structural lesion. Twenty (21%) patients had multiple potential etiologies (14 with two etiologies and 6 with three etiologies), including seventeen (17/47; 36%) with secondary hyponatremia. Etiology remained unknown in 20 (21%) participants.

Participants were followed, on average, 236 days (7.7 months; median 4.4 months [IQR: 0.8–12.5 months]). Follow up was abbreviated for those who died (2.3 versus 11.3 months, *p* < 0.001). Patients had an average of seven follow-up encounters with a mean of 42 days between encounters. Two (2/95 2%) participants withdrew due to relocation; the remainder were followed until death or study closure.

### Seizure recurrence and associated factors

Twenty-three (23/95; 24%) patients experienced at least one, and 9/23 (10%) more than one recurrent seizure during follow up. Of those who presented with new-onset seizures limited to a 24-h period prior to enrolment (*n* = 72), 21 (30%) experienced recurrent seizure during follow up. Although time to first recurrent seizure varied substantially, 14/23 recurrent seizures occurred within four months after enrolment. Death was a significant competing risk for seizure recurrence. Among participants that survived until study closure (*n* = 58), 20 (34%) experienced recurrent seizures whereas 3/37 (8%) of patients who died during follow up experienced recurrent seizure. One participant died in status epilepticus. After controlling for death, presenting in status epilepticus was associated with recurrent seizure during follow up (*p* < 0.003). Individuals who presented initially in status epilepticus experienced seizures on average later during follow up (Table 2). However, this association is likely complicated by increased frequency of recurrent seizure among individuals presenting in status epilepticus compared to individuals who did not present in status and early AED initiation after presentation. Neither an abnormal EEG nor a structural brain lesion on neuroimaging was associated with recurrent seizure (*p* = 0.205 and *p* = 0.627, respectively).

**Table 2:** Demographics and clinical characteristics of patients associated with seizure recurrence, controlling for the competing risk of death (*n* = 95)

	Median time to seizure recurrence [IQR]	*P*-*value*^*a*^
Gender		0.960
Male (*n* = 13)	3.1 [1.2–9.3]	
Female (*n* = 10)	3.3 [0.5–14.6]	
Presenting seizure severity (*n* = 89)		**< 0.01**
Status epilepticus (*n* = 17)	3.1 [1.2–9.3]	
Not status epilepticus (*n* = 4)	1.5 [0.4–8.8]	
Evidence of seizure focality		0.339
Yes (*n* = 14)	3.1 [1.2–9.3]	
No (*n* = 9)	1.1 [0.5–8.8]	
WHO clinical stage		0.334
I or II (*n* = 4)	6.4 [1.4–13.5]	
III or IV (*n* = 19)	3.1 [0.9–9.3]	
cART use		0.105
Yes (*n* = 8)	1.4 [0.3–6.3]	
No, defaulted (*n* = 4)	2.8 [1.5–10.0]	
No, never (*n* = 11)	8.6 [1.2–15.9]	
CSF collected for analysis		0.845
Yes (*n* = 20)	3.5 [0.7–12.6]	
No (*n* = 3)	2.6 [2.2–3.7]	
CNS opportunistic infection		0.788
Yes (*n* = 5)	9.3 [8.7–15.9]	
No (*n* = 18)	5.0 [0.9–8.6]	
Unknown seizure etiology		0.230
Yes (n = 3)	1.2 [0.03–1.8]	
No (n = 20)	3.8 [1.1–12.6]	

Data is presented as median (IQR). ^a^Gray’s test of cumulative incidence functions. *WHO*, World Health Organization. *cART* combination antiretroviral therapy. *CSF* cerebrospinal fluid. *CNS* central nervous system

Twenty-two (22/95; 23%) participants initiated an AED. Carbamazepine was the most commonly used AED (*n* = 21), followed by phenobarbital (1) and valproic acid (1). Twelve (13%) initiated an AED during hospitalization and continued it during follow up. Of those that began an AED during hospitalization, 6/12 (50%) experienced recurrent seizure during follow up. A post hoc analysis indicated that AED initiation during hospitalization was not associated with presenting with status epilepticus (*p* = 0.251), multiple seizures prior to presentation (*p* = 0.548), advanced HIV infection (*p* = 0.651), CD4^+^ T-cell count at enrolment (*p* = 0.929), or seizure focality (*p* = 0.129). However, it is likely that this analysis was underpowered. Of those not on an AED at discharge (*n* = 83), 17 (20%) experienced recurrent seizure. Eleven (11/17, 65%) initiated an AED as a result. Six participants did not initiate an AED despite recurrent seizure as providers considered their seizures to be provoked by alcohol withdrawal (3), the participant refused (2), or the participant died (1). Few of the patients were seen by health care providers outside of the AIDC, therefore, AEDs were typically managed in this setting. Of those prescribed AEDs, five (23%) stopped their AED either because they ran out of drug or chose to stop independently without consulting a provider. One stopped after consulting a provider.

### Risk of death and associated factors

**Fig. 1:**
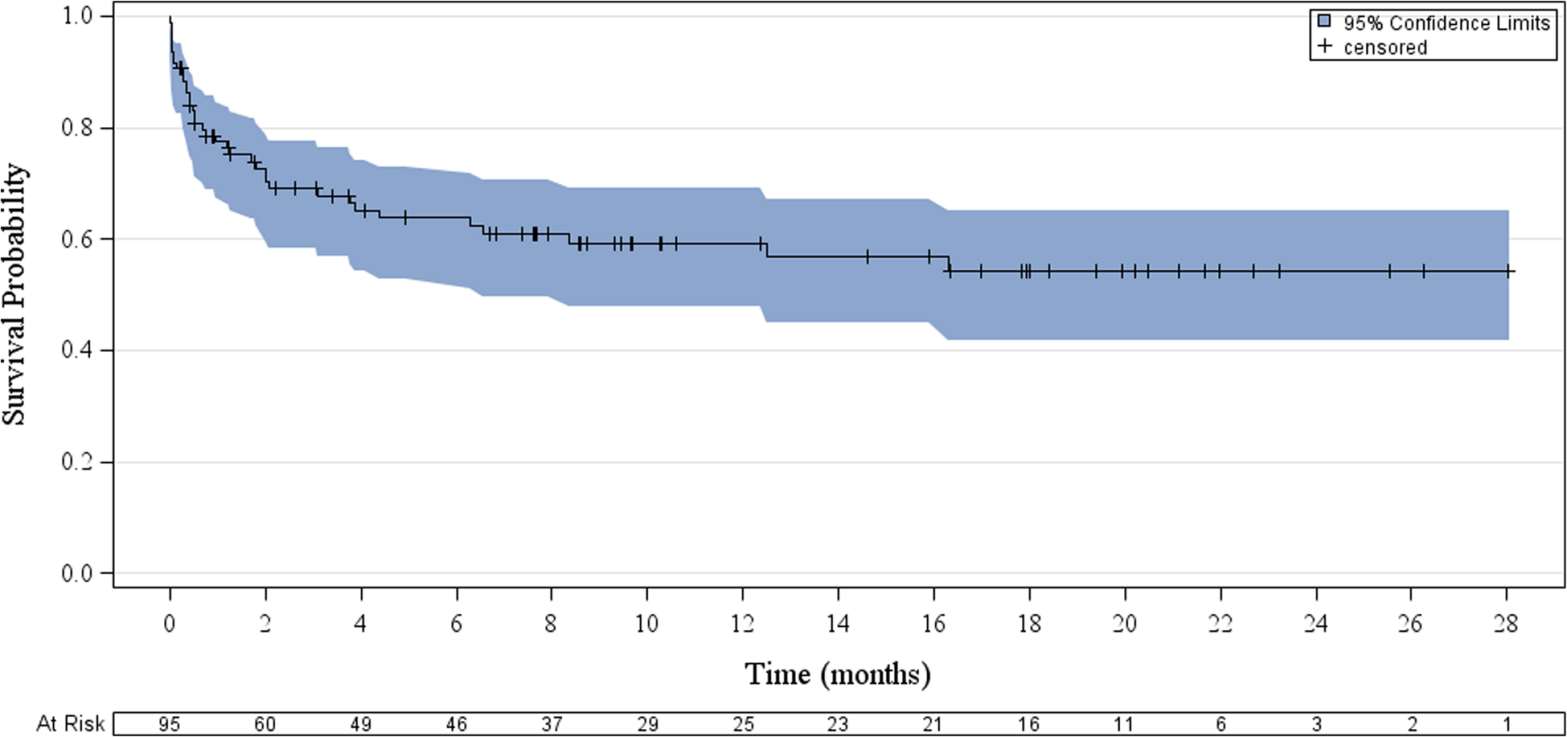
Mortality after new-onset seizure in HIV^+^ Zambian adults

**Fig. 2:**
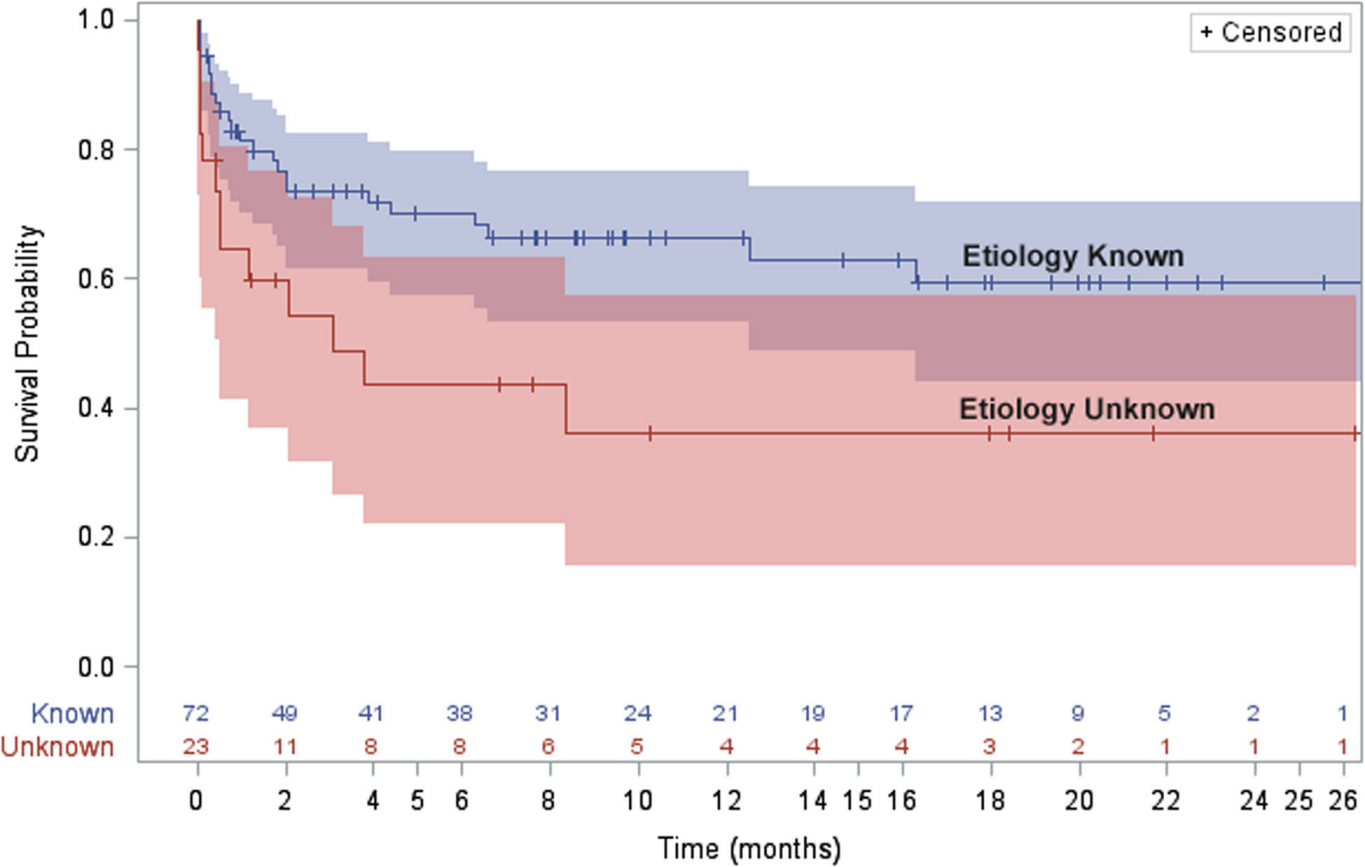
Mortality after new-onset seizure by etiology among HIV^+^ Zambian adults

During follow up, 37 (39%) patients died (Fig. 1). Eighteen (18/95, 19%) participants did not survive their initial hospitalization. Twenty-two (59%) were not on cART at the time of death. Of those on cART at the time of death, 9/15 had been on it less than a year. Among those who survived hospitalization yet died during follow up, median time to death after enrolment was 2 months (IQR: 1.3–6.3 months). Death during initial hospitalization or during follow up was associated with WHO Stage III or IV HIV infection at enrolment (HR: 19.1 [95% CI: 1.1–333.4], *p* = 0.01) (Table 3). Death was also associated with an unknown seizure etiology (HR: 2.2 [95%CI: 1.1–4.4], *p* = 0.025, Fig. 2). Death was not associated with an abnormality on EEG or neuroimaging (*p* = 0.173 and *p* = 0.56, respectively). Among participants with a full etiologic workup, including CSF DNA PCR (*n* = 61), the trend for this association remained but it was no longer significant (HR: 2.1 [95% CI: 0.8–5.3], *p* = 0.129). In a post hoc analysis, no demographic or clinical variables, including CSF collection, were associated with an unknown seizure etiology.

**Table 3:** Individual characteristics at enrollment and their association with death (*n* = 95)

Characteristic	Hazard Ratio [95% CI]	*P-value* ^*a*^
Gender, female	0.9 [0.5–1.7]	0.745
Age, mean	1.01 [0.98–1.05]	0.383
WHO HIV clinical stage III or IV^b^	19.1 [1.1–333.4]	0.01
cART use at enrollment	1.5 [0.8–2.9]	0.240
Karnofsky score < 50 at enrollment	1.8 [0.9–3.8]	0.125
Seizure severity at enrollment		
Brief, Single	Referent	0.173
Multiple, prolonged	1.7 [0.7–3.9]	
Status epilepticus	2.6 [0.96–7.3]	
CNS opportunistic infection	0.9 [0.4–2.2]	0.889
Unknown etiology	2.2 [1.1–4.4]	0.025
Among those with CSF (*n* = 61)	2.1 [0.8–5.3]	0.129

Data is presented as the hazards ratio for the listed characteristic versus the null with a hazards ratio of 1.0 indicating no difference. *CI* confidence interval, *WHO* World Health Organization, *cART* combination antiretroviral therapy, *CNS* central nervous system, *CSF* cerebrospinal fluid

^a^Log-Rank Tests ^b^As compared to WHO clinical stage I or II

During follow up, six (6%) patients were readmitted to UTH for non-seizure related illness. Thirty-nine participants not on cART at enrolment initiated treatment (39/65; 55%); average time to initiation was 61 days. Twenty-two (23%) participants never started cART. One participant declined cART despite having a CD4^+^ T-cell count < 250 cells/mm^3^, while the remaining 22 died before cART could be initiated. Time to cART initiation was not associated with a diagnosis of AIDS at study enrolment (*p* = 0.30). During follow up, four patients on cART at enrolment (4/30; 13%) defaulted treatment. AIDC clinical providers switched eight (8/69; 12%) participants to different cART regimens due to clinical or virologic treatment failure.

## Discussion

Assessment of seizure recurrence among HIV-positive Zambian adults after new-onset seizure is complicated by mortality, despite good functional status at baseline. We followed all patients for an average of 7.7 months and tracked survivors for 11.3 months. During that time, 23 (24%) patients experienced at least one recurrent seizure. Of those that survived to study closure, one-third had recurrent seizure. In contrast, among Nigerians seen at an outpatient HIV clinic, 13/20 (65%) developed recurrent seizures within a year of their index seizure [10]. These patients were likely healthier than the CHASE cohort as they were recruited from the outpatient setting and had a higher seizure recurrence primarily due to survival.

Death was a significant competing risk for seizure recurrence and, as a result, few predictors of seizure recurrence were identified. Initial seizure focality and severity were based on patient and witness report, which inherently limits classification using the 2017 International League Against Epilepsy seizure classification. Further, application of the International League Against Epilepsy’s practical clinical definition of epilepsy was limited by participant mortality and cART initiation, both of which likely reduced seizure recurrence during follow up [24]. A larger sample size, including adults with WHO stage I and II HIV infection, is warranted to examine recurrent seizure predictors among this extremely ill patient population. Initiating an AED in a patient with new-onset seizures has significant implications. In addition to lifestyle modifications required to ensure medication adherence, AEDs may decrease overall adherence to other medications and may adversely interact with the patient’s other medications, particularly cART [14]. While newer AEDs available in high-income settings are not associated with enzyme-induction, in resource-limited settings like Zambia, enzyme-inducing AEDs such as carbamazepine and phenobarbital continue to be the most available therapies. Additional research is needed to determine the pharmacologic and virologic effects of AED and cART co-usage [13]. Further, recognizing adverse effects associated with co-usage of AEDs and cART is imperative [25], particularly in resource-limited settings where there is a shortage of neurologists and AED use is frequently left to primary care providers with limited neurologic training.

In Phase I of the CHASE study, 39% of urban adult participants died during follow up, nearly half of which occurred during their initial hospitalization. Our mortality rate is similar to a 10-year primarily retrospective inpatient review of HIV-positive patients with new-onset seizure in Cameroon [26], yet lower than inpatient data reported from developed countries prior to the availability of cART [4]. CHASE mortality was associated with advanced infection, consistent with a prospective study of 17 patients in Spain [7]. Death was also associated with failure to identify an underlying seizure etiology at presentation. Diagnostic limitations, such as an inability to obtain regular CD4^+^ counts and viral loads, limited our ability to confidently identify immune reconstitution syndrome (IRIS) in participants. While 15/37 participants who died were not on cART at the time of death, 11/22 participants on cART had been on it less than a year.

The generalizability of the outcomes and risk factor data relying on CSF analysis, EEG, and neuroimaging may be inadequate, particularly where diagnostic measures are scarce and, when available, limited by cost or capacity. While we employed all the available laboratory and imaging to assess seizure etiology in our setting, a negative CSF PCR assay does not guarantee the absence of infection, particularly for conditions like tuberculosis, where testing sensitivity is less than ideal [15]. Unfortunately, not all patients consented to a lumbar puncture, therefore, to improve the external validity of our findings, we performed a sensitivity analysis with only patients who had CSF available to assess the impact of lumbar puncture refusal on findings as prior data from this setting required CSF analysis for inclusion [27]. Because of this, we were unable to apply select clinical terms, such as new-onset refractory status epilepticus (NORSE), to our study population. Similarly, unlike prior studies in this setting, patients with a family history of epilepsy or a personal history of head trauma were eligible for inclusion [8, 10]. Given the documented challenges associated with obtaining consent for lumbar puncture in many resource-limited settings, efforts to improve procedure understanding and uptake may be warranted [28–30].

Whether more expedited HIV diagnosis and cART initiation could have decreased the high mortality rate in this population is unclear. Among CHASE participants, the average time to cART initiation was 61 days and this was likely abbreviated by staff advocating for follow up visits at AIDC. While earlier initiation of cART among patients with OIs decreases mortality [31, 32], a diagnosis of AIDS was not associated with earlier cART initiation in our patient population. Twenty-two participants survived their initial hospitalization yet died before cART was initiated, despite meeting national eligibility criteria to initiate treatment. As resource-limited countries implement universal treatment access irrespective of WHO stage or CD4^+^ T-cell count, the number of HIV-positive individuals eligible for cART will grow and delays in cART initiation may increase substantially due to an overwhelmed primary health system. Efforts dedicated to shortening these treatment delays are imperative, particularly for extremely ill patients.

## Conclusions

New-onset seizure among HIV-positive Zambian adults is associated with a nearly 40% mortality rate despite good functional status prior to presentation. Patients with advanced HIV infection or an unknown underlying seizure are most likely to die during follow up. Death is a significant competing risk factor for the development of epilepsy and, as a result, we were unable to identify significant risk factors for future seizures. Among survivors, 24% experience recurrent seizures often less than a year after the index seizure.

## Abbreviations

AED: Antiepileptic drug
AIDC: Adult Infectious Disease Centre
AIDS: Acquired immunodeficiency syndrome
cART: Combination antiretroviral therapy
CHASE: Cohort for HIV-Associated Seizures and Epilepsy
CI: Confidence interval
CNS: Central nervous system
CSF: Cerebrospinal fluid
DNA PCR: Deoxyribonucleic acid polymerase chain reaction
EBV: Epstein-Barr virus
EEG: Electroencephalogram
HIV: Human immunodeficiency virus
IQR: Interquartile range
IRIS: Immune reconstitution inflammatory syndrome
MSU BIRB: Michigan State University’s Biomedical Institutional Review Board
OI: Opportunistic infection
UNZA BREC: University of Zambia Biomedical Research Ethics Committee
UTH: University Teaching Hospital
VDRL: Venereal disease research laboratory
WHO: World Health Organization
